# Using farmers' local knowledge of tree provision of ecosystem services to strengthen the emergence of coffee-agroforestry landscapes in southwest China

**DOI:** 10.1371/journal.pone.0204046

**Published:** 2018-09-20

**Authors:** Clément Rigal, Philippe Vaast, Jianchu Xu

**Affiliations:** 1 Key Laboratory for Plant Diversity and Biogeography of East Asia, Kunming Institute of Botany, Chinese Academy of Sciences, Kunming, Yunnan, China; 2 University of Chinese Academy of Sciences, Beijing, China; 3 World Agroforestry Centre (ICRAF), East and Central Asia Regional Office, Kunming, China; 4 CIRAD, UMR Eco&Sols, Université Montpellier, Montpellier, France; 5 World Agroforestry Centre (ICRAF), Vietnam Country Office, Southeast Asia Regional Program, Hanoi, Vietnam; Chinese Academy of Forestry, CHINA

## Abstract

Intensive monoculture coffee farms quickly expanded in Yunnan Province in the 1990’s and 2000’s. In 2012, local authorities in Pu’er and Xishuangbanna Prefectures, the main coffee producing centre in the province, initiated a large-scale conversion program of these farms towards coffee-agroforestry systems to promote “ecologically-friendly coffee”. Shade tree inventories and household interviews were conducted in these two prefectures to characterize coffee farms and the Local Ecological Knowledge (LEK) of farmers on the provision of ecosystem services by associated tree species. This study on newly emerging coffee farming systems revealed a high level of tree species diversity at both farm and landscape levels despite the previous dominance of intensive coffee monoculture and the large-scale distribution of a limited number of shade tree species by the government. 162 tree species were encountered during farm inventories, out of which the community of coffee farmers was able to rank 30 against 9 ecosystem services and disservices. This study reveals that this LEK is a type of hybrid knowledge that still relies mostly on traditional knowledge of tree species combined with experience acquired from newly-implemented coffee-agroforestry practices. This study also pointed out knowledge gaps regarding the impact of mature trees on coffee yield, coffee quality and pest control. The participatory approach resulted in the identification of non-promoted species with a high potential to provide locally relevant ecosystem services in coffee-agroforestry systems. These results lead to the upgrade of an online tool (www.shadetreeadvice.org) which allows extension services generating lists of recommended shade tree species tailored to the local ecological context and individual farmers’ needs. This tool will benefit farmers’ livelihood, support landscape health and contribute to the sustainability of the emerging Yunnan coffee agriculture sector.

## Introduction

Yunnan Province, of Southwest China, experienced rapid growth in coffee production from the 1990’s onwards. In 2014, Chinese production of green coffee reached 118,000 tons, becoming the 12^th^ largest coffee producing country in the world [1]. Yunnan Province now accounts for 98% of China’s coffee production [2]. The prefectures of Pu’er and Xishuangbanna, within Southern Yunnan, are the most important coffee-growing areas. These two prefectures were historically renowned for tea production. Coffee farms, typically between 900 to 1300 m above sea level, are now present alongside traditional tea estates.

Chinese farmers adopted coffee farming practices based on intensive management techniques introduced from Central America. These include propagating Catimor, a dwarf high-yielding coffee cultivar, with resistance to leaf rust disease (*Hemileia vastatrix*). Typically, full-sun coffee management strategies were combined with large fertilizer inputs to produce high yields. Shaded coffee was uncommon, primarily implemented by large international businesses in demonstration farms or by a few local farmers who targeted the emerging eco-friendly specialty coffee market. Full-sun monoculture coffee was by far the prevailing strategy, despite evidences of soil degradations and a long-term decline in yields in these systems [3], and a growing body of experimental studies highlighting the benefits of using shade trees. Indeed, shaded coffee has lower inter-annual variation in yields [4]. Fruit, timber or other shade tree products can supplement coffee income, buffering the impact of coffee price volatility [5]. Agroforestry systems also supports higher biodiversity [6], improves nutrient cycling [7, 8] and gives partial protection against climatic hazards [9, 10]. Furthermore, in sub-optimal conditions, shade trees can create beneficial microclimates, increasing coffee yield and improving coffee quality [11, 12]. Nonetheless, as a general rule, the benefits for yield and quality decrease when growing conditions improve, due to shade trees competing with coffee trees for light, water and nutrients [13].

In 2012, after a similar campaign for promoting the use of shade trees in tea estates, the government began to encourage the large-scale production of “ecologically-friendly coffee” by distributing free shade tree seedlings to coffee farmers. The distribution of a set of both indigenous and exotic shade tree species, among which a few trees of economic importance such as the Macadamia nut tree (*Macadamia integrifolia)*, illustrates the trade-off between biodiversity conservation and economic objectives. There has been a large-scale transition from monoculture coffee to shaded coffee cultivation in both Pu’er and the neighbouring prefecture of Xishuangbanna following this campaign. Nonetheless, Sayer et al [14] recommended shifting from purely top-down engineered strategies towards bottom-up solutions to address more effectively landscape challenges. Coe et al [15] highlighted that promotion of agroforestry practices can achieve better results by taking into account fine scale variations of contexts when recommending shade tree species, rather than recommending a fixed set of a few tree species.

Scientific data regarding positive and negative externalities arising from shade trees, referred to as ecosystem services (ES) and disservices (ED) [16], is lacking to thoroughly select a list of locally relevant shade tree species to promote in coffee farms in Yunnan Province [2]. In this context, local experts should be identified [17] and their Local Ecological Knowledge (LEK) used to identify suitable tree species and guide future research [18]. Studies investigating LEK have confirmed that it is highly reliable and advocated for its greater integration into policy recommendations and conservation programs [19, 20]. Furthermore, the comparison of farmers’ possession of LEK according to gender, ethnicity and farming practices can help refining our understanding of factors underpinning this LEK and improving policy recommendations [16, 21, 22]. In the present study, it can be expected that farmers with mature coffee-agroforestry systems have a rich experience and can put the list of tree species promoted by local authorities into perspective. Farmers from ethnicities traditionally settled in mountainous areas might be better acquainted with indigenous tree species from tropical montane forests, and therefore better able to point out indigenous species suitable for coffee-agroforestry systems. Additionally the comparison of LEK according to gender could reveal discrepancies in perceptions reflecting the division of responsibilities at the farm level. As such and with a concerted effort to involve farmers in the decision-making process for shade tree selection and management, a participatory approach based on farmers’ rankings has recently been developed to explore coffee farmers’ LEK regarding shade tree species and which ES and ED they can provide in coffee farms [18, 23, 24].

In this study, we first document coffee farming systems and their shade tree biodiversity at the farm and landscape levels, in view of the recent establishment of coffee as a commodity crop and the even more recent large-scale conversion from coffee monoculture towards shaded systems. Through a participatory approach based on van der Wolf et al [23], we document farmers’ LEK regarding shade tree species and their provision of ES and ED in coffee landscapes. We hypothesise that species promoted by the government are perceived favourably by farmers, especially by those most experienced in coffee-agroforestry practices. Second, we expect to find differences in farmers’ LEK according to gender. Last, we hypothesise that coffee farmers from mountain ethnicities would rank indigenous tree species higher than would farmers from lowland ethnicities.

## Materials and methods

### Compliance with ethical standards

Our study conformed to the current laws of China. All necessary permits were delivered by the Kunming Institute of Botany and local government bodies before field work. The ethics committee of the World Agroforestry Centre (ICRAF) reviewed and approved the methodology. All selected farmers gave their prior informed consents before starting interviews.

### Study sites

Fieldwork was conducted in Pu’er and Xishuangbanna Prefectures (22.80N - 100.97E / 22.00N - 100.78E), of Southern Yunnan, China. These prefectures have a sub-tropical climate and an annual average temperature of 19°C. Annual rainfall reaches 1400 mm, of which nearly 80% occurs during the rainy season from May to September. Mountains dominate the region, with elevations ranging from 340 to 3160 meters as it rises from mainland Southeast Asia to the Eastern Himalayas [25]. Areas below 800m elevation support tropical seasonal rain forests, tropical montane rain forests are found between 800m and 1200m high, and tropical montane evergreen broad-leaved forests are found at higher elevations [26, 27]. This succession of ecosystems along the elevation gradient harbours an exceptionally high biodiversity [26].

The landscape was traditionally cultivated with swidden-fallow practices [28]. From 1965 onward, changes in land-use rights and market economy led to the conversion of swidden-fallow fields and secondary forests to perennial cash crops, such as tea and rubber plantations [28]. In the early 1990’s, economic liberalization and the establishment of international buyers in Yunnan Province provided market opportunities for coffee production. Government-owned model farms began cultivating this new cash crop and distributing coffee seedlings to neighbouring farmers. Coffee farms spread between 900 and 1300m elevation, where climate is most suitable for coffee growing. They replaced perennial fields and patches of secondary tropical montane rain forest. By the early 2000’s, farms were being privatized and coffee farming kept expanding, driven by smallholder farmers and large commercial companies. Smallholder farmers typically manage 0.5 to 1.5ha of coffee plantations. Coffee represents their main source of revenues in the farm. Most of them possess small post-harvest treatment stations for wet processing, a necessary condition for complying with the certification schemes required by large agri-businesses in recent years. From spring to fall, when coffee does not require heavy labour inputs, they complement their incomes with temporary jobs outside of the farm such as construction jobs, especially important in periods of low coffee prices. Coffee companies own long-term leases for lands ranging from ten to several hundred ha. Workers typically live on farm in villages built by companies and manage plots of 0.8 to 1.2ha. By 2012, Pu’er and Xishuangbanna Prefectures had 95,000 ha under coffee cultivation, making them the main regional centres for coffee production in Yunnan according to governmental statistics [29].

The Catimor cultivar accounts for around 90% of local coffee plants, with the other 10% being two ‘traditional cultivars’, namely Bourbon and Typica [30]. Until 2012, all plantations (bar some demonstration farms) relied on intensive management practices, with high planting densities of unshaded coffee (5500 plants ha^-1^) and high inputs of mineral fertilizer (often exceeding 200 kg N ha^-1^ yr^-1^). Fungicides were seldom used, due to the leaf rust resistance of Catimor and to the absence of other major diseases. The white stem borer, *Xylotrechus quadripes*, is present, but cannot be effectively controlled by chemical inputs [31]. Preliminary interviews with governmental sources indicated that local authorities began free distribution of shade tree seedlings in 2012. This lead farmers to converting their full sun coffee to shaded coffee systems. However, introducing shade trees did not necessarily lead to eco-certifications.

### Data collection

In April 2016, inventories of shade tree species were conducted in 29 coffee farms (S1 Appendix). All the trees in coffee plantations with a diameter at breast height ≥ 5cm were systematically identified to the lowest taxonomic level possible. Abundance was estimated through rapid appraisal (visual assessment) combined with farmers’ saying. These farms were selected to include plantations representative of both young (≤15 year) and more mature trees (>15 year) and both light shaded (≤20%) and medium shaded (>20%) coffee agroforestry farms. Our sample also included 3 demonstration farms with high levels of tree diversity. All these farms were located along a latitudinal gradient (North 22.00 to North 23.26) in order to encompass the diversity of shade tree species likely to be found in the study area. Tree identification cards depicting tree characteristics along with their local names, were created for tree species that scored higher than 1% in a rank-abundance analysis and were seen in more than 20% of the coffee farms.

Between May and August 2016, coffee farmers were interviewed to document their local knowledge about shade tree species and their impacts on locally important ES & ED, following the methodology of van der Wolf et al [23]. 122 farmers were selected from the list of 4C-certified suppliers working with Nestlé Company. Furthermore, additional farms that we identified during our visits or referred to us by local authorities, which were not 4C-certified but nonetheless supported diverse and mature agroforestry systems, were also included in the sample. This way, 21 additional farmers with rich experience in agroforestry systems were added to the sample [17]. In total, 143 coffee farmers whose agroforestry systems had been established four or more years ago were interviewed. It was made clear to farmers that the results of this independent research would not impact their support either by the government or Nestlé. Farmers with mature shade trees (>15 year), medium shaded systems (>20%) and high tree species richness (>15) were expected to have richer first-hand agroforestry experience and were therefore included in priority in the sample to increase the quality of the LEK results as proposed by Davis and Wagner [17]. We tried to have a gender-balanced sample of respondents, and to interview both farmers of ethnic minority groups traditionally inhabiting mountainous areas (Bulang, Hani, Lahu, Wa, Yi, and Zang) and Han and Dai farmers, who traditionally inhabit valleys [32]. Each farmer was asked to list the shade tree species present in their farm, aided by the tree identification cards. Any additional tree species that were named during this process were cross-checked with the results from previous inventories and given a new tree identification card. In total, 44 cards were produced. Interviewees were then asked to select up to 10 tree species present in their farms, with which they were the most familiar. Using the tree identification cards, tree species selected by interviewees were ranked for their performances for each of the ten locally most important ES & ED, plus one additional rank for overall personal preference. Ties were allowed in the ranking. During this exercise, farmers were asked to comment on and explain their choices. This allowed checking how reliable their rankings were and further understanding the conceptions underlying LEK on shade trees.

Locally important ES & ED were identified in a step-wise manner. A list of 28 ES & ED was derived from the literature. Through discussions with coffee workers and managers during coffee-farm inventories, we reduced this list to 24 locally relevant ES & ED. A final list was established after the completion of 30 interviews and rankings, with each respondent selecting what they considered to be the ten most relevant ES & ED. At the end of the initial set of 30 interviews, a final list was produced of the ten most frequently mentioned ES & ED.

During the interviews, socio-economic information about the respondents was collected, including gender, ethnicity, original hometown and year of arrival in the village if respondents were not local. We also gathered comprehensive background information about their coffee farms and management practices.

### Data analysis

Shade tree species inventories were analysed using the Vegan and Biodiversity R packages in R3.3.1 [33]. The species accumulation curve was plotted with its confidence interval and the total richness of shade tree species was extrapolated using a first-order Jackknife formula [33]. Rankings by respondents on their perceptions of the shade tree species performances were analysed using the BradleyTerry2 package in R2.2.11 [34]. Rankings were converted into pairwise comparisons to be fitted to the Bradley-Terry model. A separate analysis was conducted for each ES or ED. To take into account the small sample size, the model was fitted with a bias-reduced maximum likelihood. Species that were ranked less than 10 times by coffee farmers were excluded from the analysis, to ensure enough comparisons between tree species to yield statistically significant results [24]. Consequently, 30 tree species were included in the analysis. A score *α*_*i*_ and quasi-standard error were calculated for each species *i* and each ES & ED. These scores reflect the likelihood that species *i* would perform better than species *j* for the considered attribute based on Eq (1). These are comparative, rather than absolute values. For ease of comparison, scores were normalized between 0 and 1 before being uploaded to an online tool (http://www.shadetreeadvice.org) [23]. The quasi-standard errors give an indication of how many times a species was selected and an indication of consistency in the respondents’ rankings. High quasi-standard errors indicate low LEK regarding a given species. Scores were compared pairwise using a Wald test. Results were then presented and validated through focus group discussions with coffee farmers and extension agents.

$$P(Species\;i\;performs\;better\;than\;Species\;j)=\frac{\propto_{i}}{\propto_{i}+\propto_{j}}$$ (Eq 1)

where ∝_i_ is the score of species *i*

### Exploration of local knowledge

The Bradley-Terry method has previously been used to analyse rankings of tree species [18, 24]. When comparing the perceptions of respondents from distinct groups, for instance based on gender or elevation, these studies conducted separate analysis for each group and compared the resulting scores. The present study is the first to incorporate predictor variables into applications of the Bradley-Terry method to LEK. This allows comparing perceptions of respondents from distinct groups through a single analysis, thus maintaining the initial sample size. Specifically, it allows analysis of interactions between a tree species characteristic and a socio-economic attribute of respondents’ rankings. This analysis is used to answer the question: do farmers with this attribute rank tree species with this characteristic differently than do other farmers? Tree species were classified as being promoted or not by the local authorities and as indigenous species versus exotic ones. Respondents were assigned three qualitative attributes: 1) gender (M / F), 2) agroforestry system (low shade / medium shade) and 3) traditional location of ethnic groups (mountains / lowlands). The shade tree species richness on their farms was included as a quantitative attribute.

Interactions between respondent attributes and tree species attributes were incorporated into separate analyses to test the following three models:

Model A: Farmers with high tree species richness and medium shaded systems (respondent attributes) rank promoted species (tree species attributes) more highly than farmers with low species richness and low shaded systems.

Model B: Gender affects the rankings of promoted species.

Model C: Farmers from mountain ethnicities give higher ranks to indigenous species than farmers from lowland ethnicities.

### Suitability of government-promoted tree species to specific local contexts

The regional impact of government promotion of shade tree species, through the distribution of free seedlings, was quantified by comparing scores of promoted species versus non-promoted species using a Student’s T test for each ES or ED. Then, scenarios were simulated for three hypothetical coffee farmers, all located in the study area, but facing contrasting local constraints and hence adopting different strategies. These were: 1) a farmer whose land is located at high altitude and has a need for additional frost-protection by shade trees, 2) a farmer who is enhancing soil fertility through the addition of shade trees to reduce fertilizer inputs, and 3) a farmer whose goals are to cut input costs (fertilizers) and enhance income diversification with shade trees. Based on the results from the Bradley-Terry analysis and on the tree selection tool developed by van der Wolf et al [23], scores were attributed to 30 tree species for each scenario. In each scenario, species with the highest scores are those perceived to have the highest performance for the selected set of ecosystem services, and so would be the species recommended to farmers in comparable circumstances.

## Results

### Shade tree species inventories

**Fig 1:**
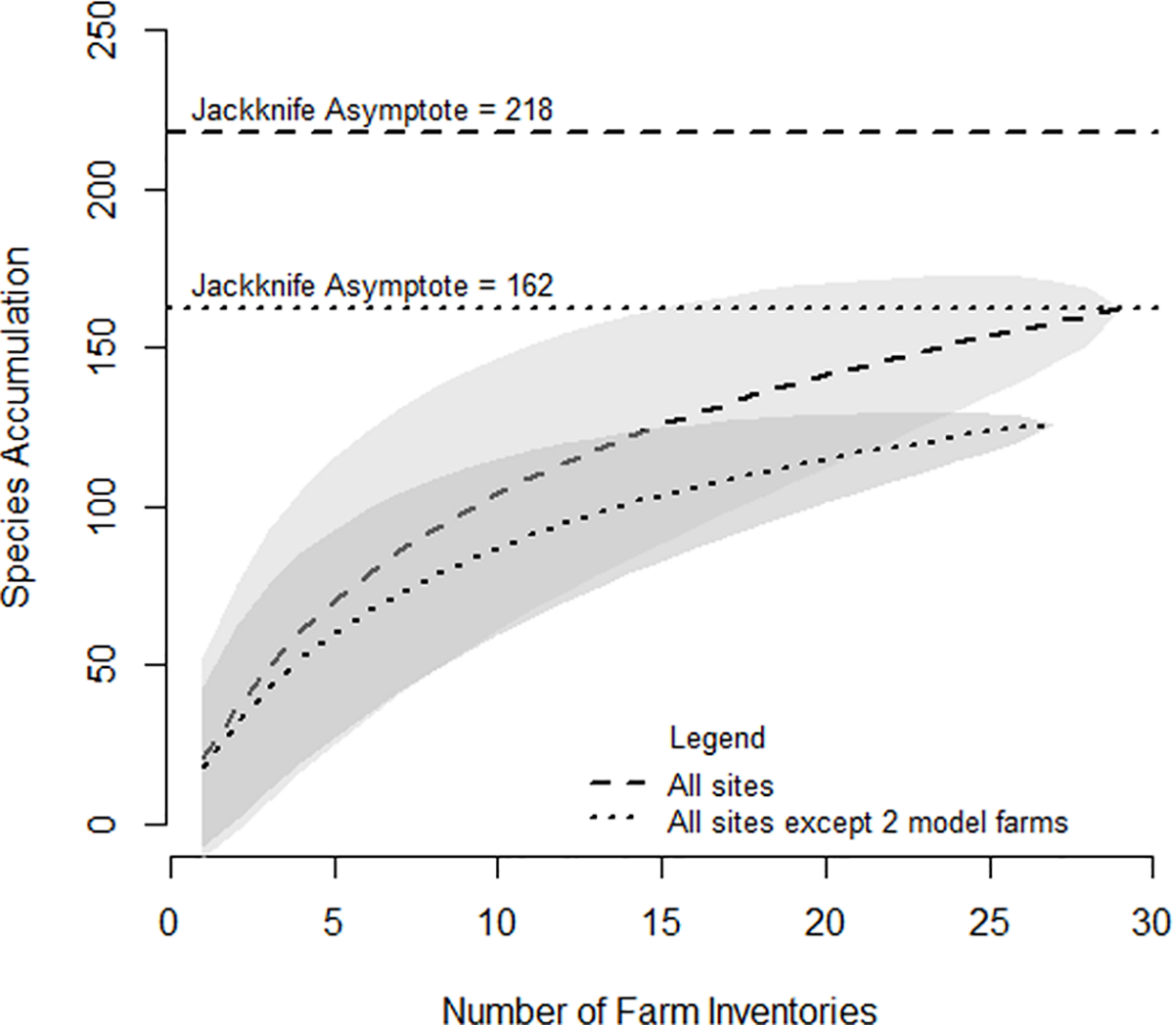
Species accumulation curves and 1st order Jackknife asymptotes with (dashed line) and without (dotted line) data from 2 demonstration farms. Grey areas represent the 95% confidence intervals.

During initial inventories in coffee farms, 162 shade tree species were encountered, of which two could not be identified, five were identified up to genus level and the other 155 were identified to species level. Across the 29 inventoried coffee farms, the Shannon index ranged from 0.00 to 3.42, with a mean value of 2.22. The effective number of species, calculated as the exponential of Shannon entropy [35], ranged from 0 to 30.57 species, with a mean value of 9.21 species. The Simpson index varied from 0.00 to 0.96, with a mean value of 0.79. Further details are provided in S2 Appendix. First-order Jackknife formula was used to extrapolate the total richness of shade tree species in the study area. This led to an estimated value of 218 tree species (Fig 1), according to which 74% of all shade tree species were encountered during our inventories. When two non-representative demonstration farms which each exhibited a high and non-representative level of tree species diversity were removed from the analysis, the extrapolated number was reduced to only 162 tree species.

**Fig 2:**
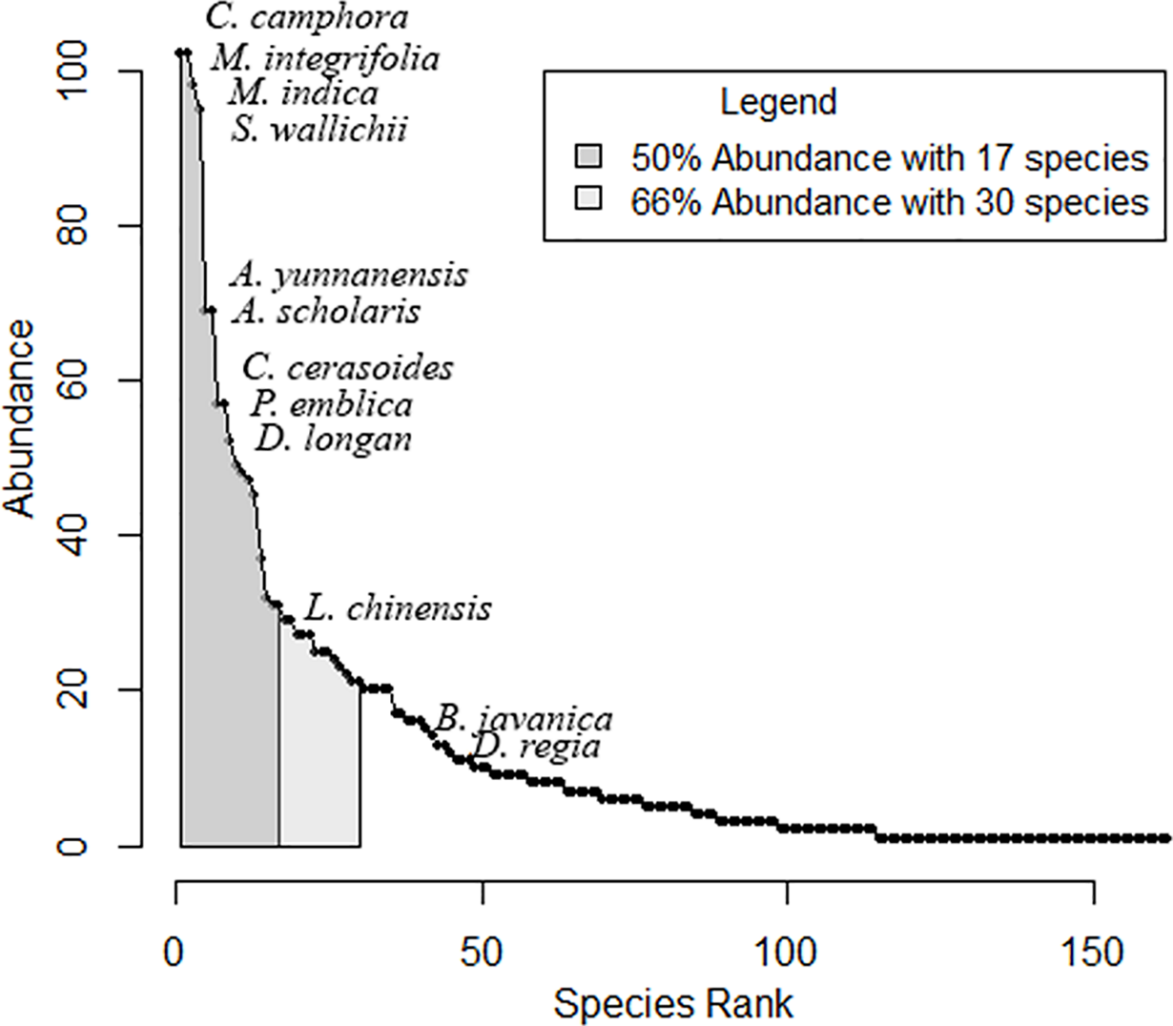
Rank-abundance curve from coffee farm inventories. The 9 most abundant species and the promoted species are represented on the curve.

Only 17 tree species accounted for more than half (51%) of all the trees inventoried (Fig 2). 84 species were only encountered in one or two coffee farms. The nine government-promoted species (Table 1) represented 27% of all the trees inventoried. The two most abundant non-promoted species were *Mangifera indica* and *Schima wallichii*. The latter is an early successional species that has spread through natural regeneration.

**Table 1:** List of the 30 shade tree species ranked by coffee farmers and ecosystem services reported by farmers.

Latin Name	Chinese Name	Promotion Status	Indigenous (I) / Exotic (E)	Ecosystem Services
*Albizia kalkora*	山合欢		I	N-fixation
*Aporosa villosa*	毛银柴		I	Fruit
*Aporosa yunnanensis*	滇银柴		I	Fruit
*Artocarpus heterophyllus*	波萝蜜		E	Fruit
*Betula alnoides*	西南桦		I	Timber
*Bischofia javanica*	重阳木	Promoted	E	Urban landscaping, timber
*Castanopsis calathiformis*	枹丝锥		I	Firewood
*Cerasus cerasoides*	云南樱桃	Promoted	I	Fruit, Ornamental
*Cinnamomum camphora*	香樟	Promoted	I	Urban landscaping, Medicine
*Delonix regia*	凤凰木	Promoted	E	Urban landscaping, Ornamental
*Dimocarpus longan*	龙眼	Promoted	I	Fruit
*Diospyros kaki var*. *silvestris*	野柿		I	Fruit
*Eurya groffii*	岗柃		I	Shade
*Ficus hispida*	对叶榕		I	Shade
*Alstoniae scholaris*	灯台树	Promoted	I	Urban landscaping, Medicine
*Leucaena leucocephala*	银合欢		E	N-fixation
*Litchi chinensis*	荔枝	Promoted	I	Fruit
*Litsea sp*.	木姜子		I	Shade, Fruit
*Macadamia integrifolia*	澳洲坚果	Promoted	E	Fruit
*Mallotus tetracoccus*	四籽野桐		I	Shade
*Mangifera indica*	芒果	Promoted	I	Fruit
*Melia azedarach*	苦楝		I	Seeds
*Michelia baillonii*	合果木		I	Timber
*Musa balbisiana*	芭蕉		I	Fruit
*Musa basjoo*	象腿焦		E	Fruit
*Phyllanthus emblica*	余甘子		I	Shade, Fruit
*Psidium guajava*	番石榴		E	Fruit
*Schima wallichii*	西南木荷		I	Timber
*Syzygium szemaoense*	思茅蒲桃		I	Shade, Fruit
*Toona ciliata*	红椿		I	Timber

### Main characteristics of tree species

A total of 42 tree species were selected and ranked by respondents. 30 of these species were ranked more than 10 times for each attribute and therefore included in the ranking analysis. Amongst these, 20 species were endemic to the study area. Nine species have been actively promoted by the local authorities through distribution of free seedlings, amongst which five are fruit tree species and four are species valued as ornamental trees for urban landscaping (Table 1).

### Main characteristics of interviewees

143 coffee farmers were interviewed, of whom 124 responded by ranking shade tree species against ecosystem services and disservices. 19 respondents who could not rank tree species were excluded from analysis. Of the 124 respondents who did rank tree species, 42 were women (34%) and 82 were men (66%); 60 were smallholder farmers (48%), 42 were agricultural workers (34%) and 22 were coffee farm managers (18%). 48 respondents belonged to mountain ethnicities (39%) and 76 to lowland ethnicities (61%). 103 respondents were autochthons (born in the study area) (83%) and were 21 allochthons (17%). The area of coffee farms ranged from 0.3 to 180 ha, with an average of 10 ha and a median value of 2 ha. 65 respondents (52%) worked in coffee-agroforestry farms that were classified as low-shaded and 59 respondents (48%) worked in agroforestry farms that were classified as medium-shaded. Respondents listed 4 to 32 species of shade trees per farm, reporting an average of 15 species per coffee farm.

### Tree species rankings and pairwise comparisons

**Fig 3:**
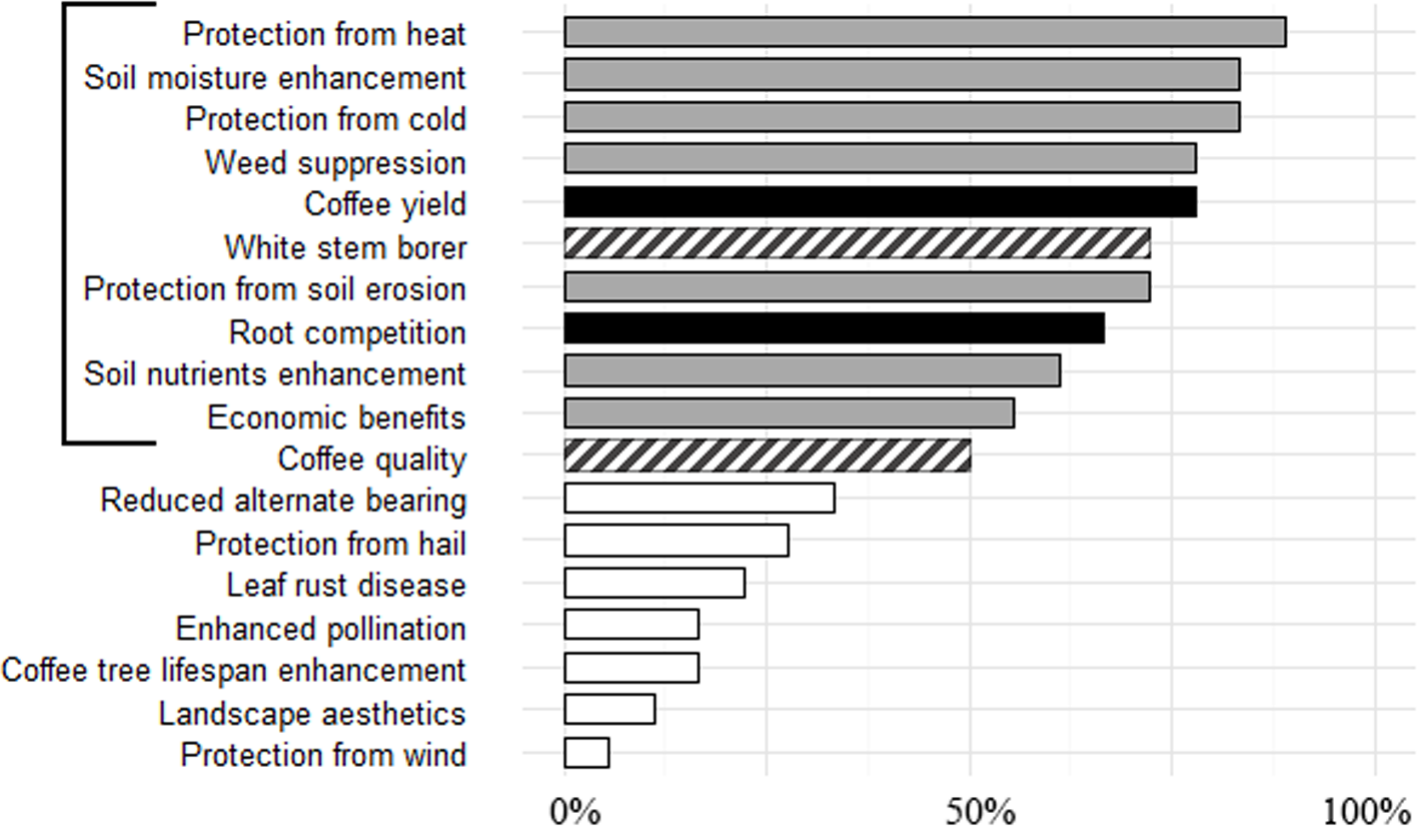
Locally relevant ecosystem services and disservices (ES & ED) after the 30 first interviews. Boxes represent the percentage of respondents for which each ES or ED was locally relevant. Grey boxes show the ES and black boxes show the ED selected for further ranking of shade tree species. The 2 striped boxes indicate ES that respondents thought were locally relevant, but for which they were unable to rank tree species.

After the initial 30 interviews, the following ten ES & ED were assessed as the most locally important: 1) protection of coffee trees against high temperatures (ES), 2) soil moisture enhancement (ES), 3) protection of coffee trees against cold temperatures (ES), 4) suppression of weeds (ES), 5) negative impact of shade trees on average coffee yield (ED), 6) control of the white stem borer (WSB) (ES), 7) protection from soil erosion (ES), 8) root competition between shade trees and coffee trees (ED), 9) soil nutrient cycling enhancement from leaf litter and N-fixation (ES) and 10) additional economic benefits from the shade trees (ES) (Fig 3). After conducting the Bradley-Terry analysis on the 124 rankings, pairwise comparisons of species’ scores showed that tree species were easiest to rank for economic benefits, weed control and protection from high temperatures (81%, 76% and 75% of all pairs were significantly distinct (p ≤ 0.05)). Trees were hardest to rank for their impact on coffee yield, root competition and enhancement of nutrient cycling (59%, 62% and 66% of all pairs were distinct). No respondents were able to rank tree species for ability to control WSB, so this ES was excluded from the analysis. Although coffee quality did not make it into the top ten ES and ED, it is interesting to note that no farmer was able to rank tree species against this important ES during the 30 first interviews. In their views, rainfalls during harvest seasons was the single most influential parameter impacting coffee quality.

### Promoted tree species

**Fig 4:**
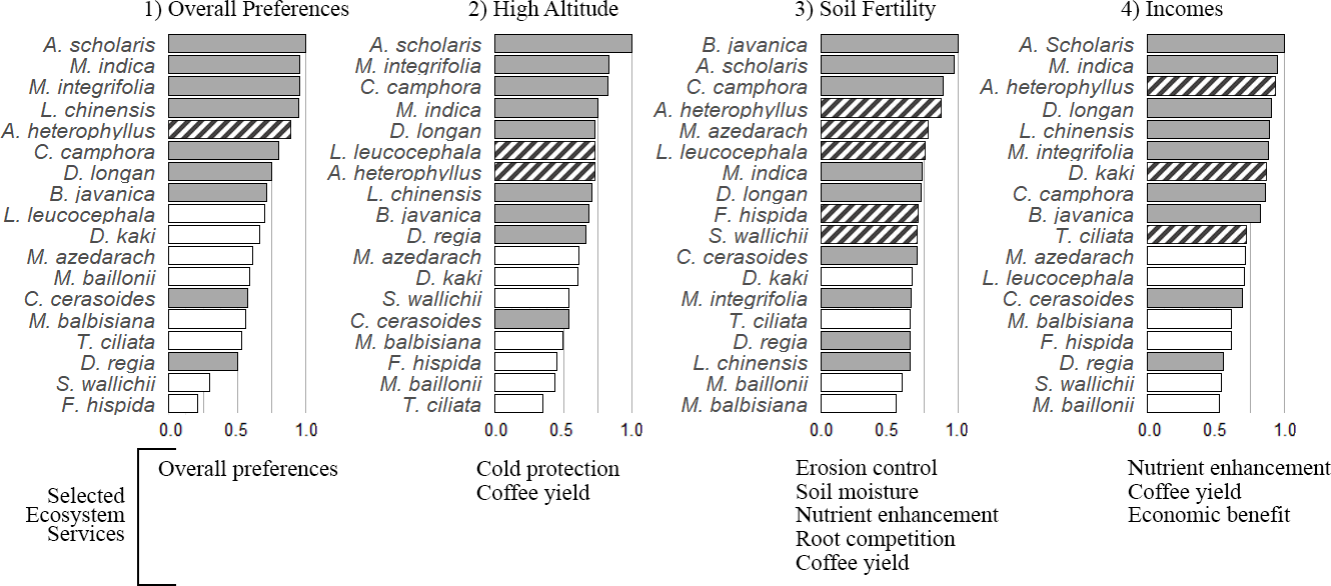
Tool outputs displaying scores for 18 shade tree species out of 30 according to 1) overall preference, and three hypothetical scenarios: 2) a high altitude farm exposed to frost risks, 3) a farm with limited or no input of chemical fertilizers, and 4) a farm where trees are primarily planted and managed for income diversification. Grey boxes indicate promoted species; striped boxes indicate non-promoted species that score highly in a specific scenario.

Promoted species were perceived to perform significantly better than non-promoted species for all attributes except one ES (nutrient cycling enhancement), and two ED, (reduction in coffee yield and root competition, Table 2). Seven of the nine promoted species were ranked in the top eight of species favoured by coffee farmers (Fig 4). *Artocarpus heterophyllus* was the only highly favoured non-promoted species. *Cerasus cerasoides* (ranked 13^th^) and *Delonix regia* (ranked 16^th^) were the only two promoted shade tree species that were ranked lower than some non-promoted species.

**Table 2:** Mean scores of promoted versus non-promoted shade tree species for individual ES & ED and overall preference according to the Bradley Terry analysis. Student T-test results highlight significant differences between groups for each ES & ED.

Species	Heat Protection	Cold Protection	Erosion Control	Soil Moisture	Nutrient Enhancement	Root Competition	Coffee Yield	Weed Control	Economic Benefit	Overall Preferences
Promoted	0.74	0.71	0.66	0.63	0.63	0.53	0.73	0.67	0.66	0.80
Not Promoted	0.48	0.38	0.39	0.46	0.51	0.53	0.62	0.44	0.33	0.42
Significance	**	****	*	.	NS	NS	NS	*	**	***

Statistical significance is indicated by ‘***’ < 0.001 / ‘**’ < 0.01 / ‘*’ < 0.05 / ‘.’ < 0.1 / ‘NS’ Non Significant.

### Coffee farming practices and ranking

Overall, farmers gave similar rankings to trees regardless of the tree species richness and degree of shade in their farms (Table 3 - Model A). However, respondents whose farms supported higher tree species richness perceived lower root competition between promoted species and coffee plants (p ≤ 0.05). Respondents whose agroforestry systems had more shade thought that promoted species were beneficial to soil moisture (p ≤ 0.05), but had a negative impact on coffee yield (p ≤ 0.05). Promoted species scored high in the list of preferred species for all respondent groups. Farmers with lower species richness and lower shade intensity on their farms had an even stronger preference for promoted species (p ≤ 0.01).

**Table 3:** Interactions between coffee farmer attributes and their rankings of promoted and indigenous shade tree species by ecosystem services and disservices. Only significant results are shown.

	Heat Protection	Cold Protection	Erosion Control	Soil Moisture	Nutrient Enhancement	Root Competition	Coffee Yield	Weed Control	Economic Benefit	Overall Preferences
**Model A**										
Promoted Spp. × Spp Richness (†)						0.01 *				-0.01 **
Promoted Spp. × Medium-Shade AFS				0.09 *			-0.13 *			-0.12 **
** Model B**										
Promoted Spp. × Gender [M]	-0.07 *				0.10			0.11 **	0.06	0.11 *
** Model C**										
Indigenous Spp. × Mountain Ethnicity					-0.13 *	-0.14 *	-0.19 **		0.12 ***	

Statistical significance is indicated by ‘***’ < 0.001 / ‘**’ < 0.01 / ‘*’ < 0.05 / ‘.’ < 0.1.

(†) refers to quantitative variables.

### Gender, ethnicity and tree species preferences

Women were more likely than men to perceive non-promoted species as more beneficial to nutrient cycling enhancement (p ≤ 0.1). Men were more likely to perceive promoted trees to bring higher economic income (p ≤ 0.1) and showed a significant preference for these species (p ≤ 0.05) (Table 3 –Model B). Men showed higher overall preferences than women for *Cinnamomum camphora* and *C*. *cerasoides* while women ranked *Litchi chinensis* and *Leuceana leucocephala* higher than men did (S3 Appendix).

Autochthon farmers from mountain ethnicities expected greater economic benefits from indigenous species than did farmers from lowland ethnicities (Table 3 –Model C). Farmers from mountain ethnicities gave high rankings for economic benefits to the indigenous species *Alstoniae scholaris*, *Toona ciliata*, *Diospyros kaki*, *Michelia baillonii*, *Betula alnoides* and *S*. *wallichii* (S3 Appendix). However, they thought that indigenous species had higher negative impacts on coffee yield through below ground interactions.

### Tailored list of recommended species

Promoted species outranked most of the non-promoted species for economic gain via cutting fertilizer cost and boosting incomes through diversification (Fig 4. Scenario 4). However, in this scenario, the non-promoted *A*. *heterophyllus* and *D*. *kaki* were advised species while the promoted *C*. *cerasoides* and *D*. *regia* were among least recommended species. Although many respondents reckoned that timber from *M*. *baillonii* is in high demand, it was only ranked 25^th^ out of 30.

Among the short-list of 30 trees, tree species that gave the best financial returns were also the best in protecting coffee trees from frost (Fig 4). Strikingly, the top nine trees for frost-protection were also the nine overall most-preferred trees. These included seven trees promoted by the local authorities, plus *A*. *heterophyllus* and *L*. *leucocephala*. As explained by farmers, six of these promoted trees were evergreen species with dense canopies, which are likely to tolerate cold temperatures and buffer coffee plants underneath from cold spells.

Tree species promoted by local authorities were not necessarily the best choice for nutrient cycling, limiting root competition or sustaining coffee yield. For this set of ES and ED, only five promoted species were among the ten most recommended trees (Fig 4). The other five non-promoted species included indigenous species with low economic benefits but perceived as enhancing soil quality, mostly through leaf litter, such as *Melia azedarach*, *Ficus hispida* and *S*. *wallichii*, and *L*. *leucocephala*, identified as a Nitrogen-fixing tree by farmers in Nestlé’s demonstration farm.

## Discussion

### Tree diversity, richness and density on coffee farms

We expected to find low tree species diversity in coffee farms, due to the recent expansion of coffee areas and current adoption of coffee agroforestry practices in Yunnan Province. In fact, our tree species inventories showed that the study area supports an estimated 162 tree species. This unexpectedly high tree species diversity is likely to reflect the biodiversity of previous ecosystems in the study area. Coffee farms spread in areas used for swidden-fallow practices until the mid-20^th^ century [28], or previously dominated by secondary to mature tropical montane rain forests [36]. Song [27] recorded 156 tree species between 800 and 1400m elevation and Cao [26] noted that rank-abundant curves were characterized by long tails, indicating that the tree species diversity of tropical montane forests mostly depended on rare species. The dominance of *S*. *wallichii* and *Castanopsis* species [27, 36], also common in our tree inventories, further indicate similarities between shade trees found in coffee farms and the forest ecosystems they replaced. Last, diversity indices from these forest studies were also similar to those derived from our tree inventories.

Diversity indices from the present study can be compared to those derived from tree inventories conducted in similar intensive coffee systems, located between 800 and 1,250m elevation in Costa Rica [37]. That study recorded only 104 shade tree species in conventional farms. On the other hand, there were 19 species on average per farm in Costa Rica compared to 15 species per farm in our study area.

Tree species were unevenly distributed across our study area, with 52% of species occurring in only one or two farms. Dawson et al [38] emphasized that the prospects of conservation for low density species in the agricultural landscape are poor because these species are particularly vulnerable to being wiped out by the decision-making process of a few farmers. Low densities can also restrict pollination and reproduction. If maintenance of high coffee yield requires the thinning of shade trees, the least appreciated tree species will be the first to be felled. This is likely to reduce biodiversity by increasing the proportion of economically profitable non-native species [39].

### Gaps in local knowledge of shade trees

Agroforestry practices in coffee systems have only recently been promoted and adopted on a wide-scale across Yunnan. Still, local knowledge on shade tree species and their impacts on ecosystem services in coffee farms is already well-developed. 87% of respondents could rank tree species for at least one issue. Some level of LEK was present across all socio-economic groups, although the degree of LEK varied with agroforestry systems, gender and ethnicities.

Although coffee farmers know local tree species and their phenology, they still have limited experience of the impact of mature shade trees on coffee yield. They gave high ranks to several fruit tree species such as *M*. *integrifolia*, *L*. *chinensis* and *Dimocarpus longan*, which have dense canopies and hence have high potential to compete for light with coffee plants. Because most shade trees are young, such negative impacts may not yet have become apparent [4, 13], or farmers could be more concerned about other economic factors. Farmers with more first-hand experience in agroforestry systems did nonetheless perceive higher negative impacts of promoted species on coffee yield, than did farmers with seemingly less experience. Farmers varied widely in their assessment of root competition and nutrient cycling. This would be expected because such complex below-ground factors are the most difficult to assess [24]. They also lacked knowledge regarding the white stem borer and coffee quality. As farmers gain greater experience in cultivating coffee, their LEK is likely to be progressively enriched.

### Relevance of promoted tree species

Local stakeholders perceived the tree species promoted by the local authorities to provide the best protection against weather hazards and bring the greatest economic benefits. It appears that shade tree species were preferentially promoted when they have dense canopies and high economic returns, despite their negative impact on coffee yield. Apart from *D*. *regia*, which has only aesthetic value, all promoted species were perceived to have positive economic returns. Income can be derived from fruit production or selling saplings to the emerging market for tree landscaping in nearby fast-growing cities. Five year-old saplings are uprooted and sold for re-planting alongside the new roads and sidewalks of China’s rapidly expanding cities. Our respondents reported that *A*. *scholaris* is the “urban tree” in highest demand, followed by *C*. *camphora* and *B*. *javanica*. Income diversification is a prime motive for the selection of shade tree species by coffee farmers. Therefore, it is not surprising that farmers prefer those promoted species that produce fruit or saleable saplings. The non-promoted fruit trees, *A*. *heterophyllus* and *D*. *kaki*, were thus also highly ranked.

Farmers appeared to be most interested in gaining short-term economic benefits. They did not favour valuable indigenous timber species (*M*. *baillonii* and *T*. *ciliata*) that require long-term investment. Regional policies that emphasize forest protection and hence prohibit felling trees [40] currently discourage growing timber crops. Although there are specific conditions under which permits are granted for timber harvest, these are seldom delivered, leaving farmers doubtful of their ability to harvest timber, a concern frequently mentioned during interviews. Such problems have been reported in Kodagu, India, where policies for the protection of indigenous species in coffee estates counter-intuitively lead to decreased planting of species that will be difficult to harvest and the replacement of indigenous trees with exotic shade trees (Garcia et al, 2010). There is a need for incentive programs that support the use of indigenous timber trees and promote their planting and/or natural regeneration.

Governmental promotion of some tree species can also succeed in shaping the preferences of farmers. In Mexico, Valencia, West [41] showed that there had been little scientific foundation for the promotion of *Inga oerstediana* in coffee agroforestry systems by NGOs and government agencies 30 years ago. Nonetheless, farmers still highly prefer this species. In the present study, because farmers are new to coffee-growing and agroforestry practices, promotion and dissemination of shade trees is especially likely to shape their perceptions and actions. Indeed, farmers with seemingly low experience in agroforestry systems ranked promoted species higher than farmers with rich first-hand experience in overall preference rankings. This further support the interpretation of a collective bias driven by promotion activities from local authorities. It is therefore important to keep in mind the limits of LEK studies. Perceptions of trees can be biased or reflect partial views about provision of ES/ED by particular tree species [23]. Wherever possible, studies on LEK should thus be complemented and validated with on-farm studies of the actual interactions between shade trees and coffee trees.

### Gender, ethnicity and tree species ranking

Impact of gender on tree preferences was noticeable, with men having a stronger preference for promoted species. Women were the most likely to include N-fixing tree species in their list of favoured shade trees. No explanation could be found to substantiate either findings. Surprising gender differences in LEK have been found elsewhere; Ayantunde, Briejer [21] pointed out that women, who were responsible for cooking, could identify fewer firewood species than men. In our study, men and women had similar responsibilities in coffee farms; all participated in agricultural activities such as fertilizing, weeding, harvesting and processing crops. Further investigation of how gender is affecting LEK will require complementary tools (e.g. ATK tool), to relate rankings with socio-economic attributes [24].

We had expected that farmers from ethnicities traditionally settled in mountainous areas might exhibit preferences for some indigenous tree species commonly found at these altitudes that would not be valued in other areas. Indeed, indigenous timber trees were ranked higher by these farmers. Coffee farmers from mountain ethnicities thereby exhibited a ‘hybrid type of knowledge’ [42], combining their traditional knowledge with both first-hand experience and outside sources, in our case promotion activities from the local authorities.

### Tailored list of recommended species

We produce three separate lists of tree species that we recommend for three different farmer priorities. Our lists were very similar to that of the government, when considering strategies to diversify income sources (our third scenario), and to lower the frost risk in high-altitude farms while maintaining high coffee yield (our first scenario). The government’s list of promoted species thus appears to be well suited to coffee farmers whose priorities are primarily related to either economic benefits or protection from climatic hazards. Based on farmers’ overall preferences, and these two simulated scenarios, we recommend future promotion of *A*. *heterophyllus* and *L*. *leucocephala*.

Simulations based on soil fertility enhancement (our second scenario) would lead to the recommendation of additional species such as *M*. *azedarach*, *L*. *leucocephala*, *F*. *hispida* and *S*. *wallichii*. The promoted shade tree species boost profits at the expense of below-ground ecosystem services. On the other hand, the suggested additional species do not bring high economic benefits but are perceived to favour soil fertility. Therefore, there is no single solution for a top list of the best tree species for the entire study area. The list of shade-tree species promoted by the local authorities is a useful starting point, but it cannot address the need for complex agricultural strategies or trade-offs between economics and key ecosystem services. Rather, recommendations should be area-specific and farmer specific. The lists generated in the present study can be used as a starting point. Individual farmers should then modify them to take into account their individual LEK, their local conditions and their economic strategies.

## Conclusion

This study on newly emerging coffee farming systems in Pu’er and Xishuangbanna Prefectures revealed an unexpectedly high level of diversity amongst shade tree species, at both farm and landscape levels. At the landscape level, tree species richness in coffee farms was similar to that documented in tropical montane forests in south Yunnan. This indicates that tree species diversity has persisted despite the spread of intensive coffee monoculture and the governmental promotion of a limited number of species. These governmentally-promoted tree species were valued by coffee farmers for their perceived high economic returns and protection against environmental hazards. Favourable perceptions of promoted trees also probably reflected a collective bias driven by promotion activities by governmental extension services. However, as trees grow, there will be increased competition between some shade trees and coffee trees for light, water and nutrients. Combining the existing LEK with further research on the actual interactions between mature shade trees and coffee trees is needed in order to refine locally adapted advice on shade tree management practices, including thinning and pruning.

Currently, there appears to be a hybrid LEK that mostly relies on traditional knowledge of tree species combined with fresh experience from newly-implemented coffee-agroforestry practices. Farmers of traditional ethnic groups from mountain areas or with richer first-hand experience in coffee-agroforestry practices differed from other farms in their perceptions of shade trees and their impacts on ES and ED; they preferred some indigenous and non-governmentally-promoted species. LEK still needs to be enriched by developing experience with mature shade trees. Furthermore, this study identifies knowledge gaps regarding the impact of shade trees on coffee yield, coffee quality and control of the white stem borer. These gaps should orientate future research works to complement the existing LEK.

This participatory approach results in the upgrading of an online tool (www.shadetreeadvice.org) which complements the top-down engineered program of the government, by allowing farmers and extension services producing lists of tree species tailored to the farmers’ needs and the local ecological contexts of Yunnan Province. Further research will improve this decision support tool on tree selection and contribute to sustainable coffee management benefiting farmers’ livelihood and landscape health in Yunnan Province.

## Supporting information

S1 Appendix Methodological steps for inventorying shade trees in coffee farms and documenting the associated local ecological knowledge. (DOCX)

S2 Appendix Additional information on shade tree inventories in coffee farms. (DOCX)

S3 Appendix Comparison of tree species ranking according to gender and ethnicities. (DOCX)
