# Supplementary material for: Using farmers' local knowledge of tree provision of ecosystem services to strengthen the emergence of coffee-agroforestry landscapes in southwest China
Source: PLoS One. 2018 Sep 20;13(9):e0204046. doi: 10.1371/journal.pone.0204046 (PMC6147441; doi:10.1371/journal.pone.0204046)
Supplement: S1 Appendix — (DOCX) [file pone.0204046.s001.docx]

# S1 Appendix. Methodological steps for inventorying shade trees in coffee farms and documenting the associated local ecological knowledge

**
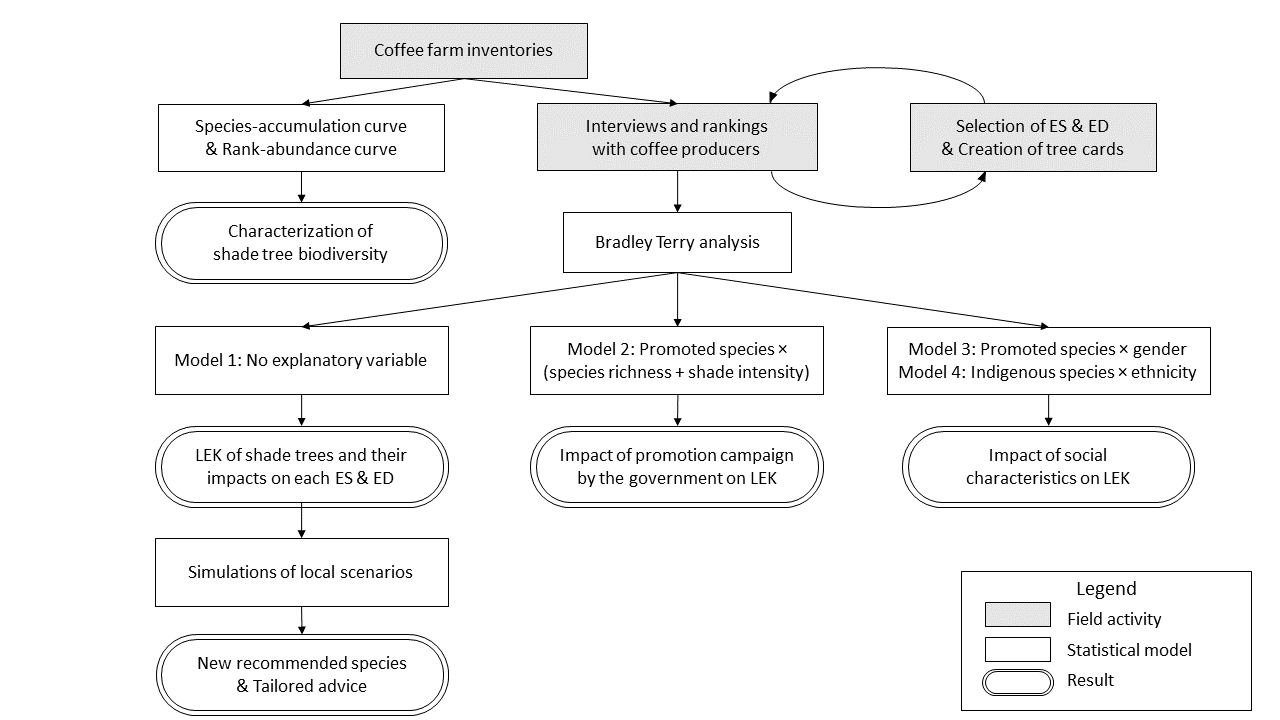
**

S1 Fig: Methodological steps
