# Supplementary material for: Using farmers' local knowledge of tree provision of ecosystem services to strengthen the emergence of coffee-agroforestry landscapes in southwest China
Source: PLoS One. 2018 Sep 20;13(9):e0204046. doi: 10.1371/journal.pone.0204046 (PMC6147441; doi:10.1371/journal.pone.0204046)
Supplement: S2 Appendix — (DOCX) [file pone.0204046.s002.docx]

# S2 Appendix. Additional information on shade tree inventories in coffee farms

Diversity indices are detailed for the 3 model farms in the table below. It can be seen that shade tree species in model farm 3 are slightly more diverse than in the 2 other model farms (S2 Table 1). Higher diversity could result from the larger area under coffee farming. However, these species were found not only in model farm 3 but also in other coffee farms, while many shade tree species found in model farms 1 and 2 were only encountered in these 2 farms.

There is a positive relationship between coffee farm area and tree species richness, as found from the respondent database (S2 Fig 1). Tree density was not estimated. Nonetheless, it is likely to be higher than 180 trees/ha, which would represent the density resulting from the average number of tree seedlings distributed by the government, plus trees originating from natural regeneration, as high levels of seedling survival were observed.

S2 Table 1: Biodiversity indices for three model farms

| Model Farm | Area (ha) | Shannon | Simpson | Effective number |
| --- | --- | --- | --- | --- |
| Farm 1 (Nestlé)  Farm 2 (Nandaohe)  Farm 3 (Aini - Starbucks) | 16  14  30 | 3.35  3.07  3.42 | 0.95  0.90  0.96 | 28.50  21.54  30.57 |


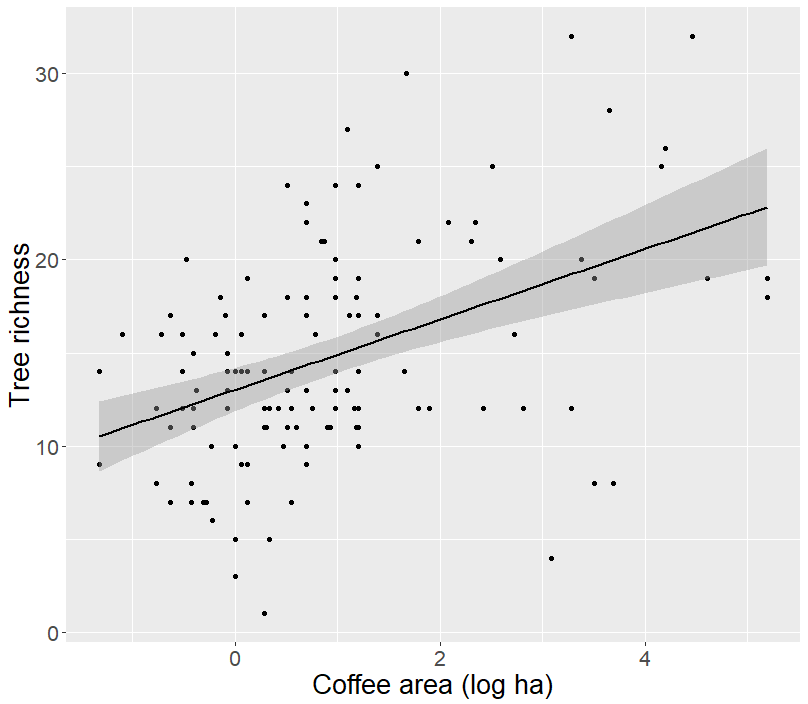


S2 Fig 1: Relationship between shade tree richness and coffee farm area based on the database of respondents

(R^2^ = 0.19, p-value < 10^-6^)
