# Supplementary material for: Using farmers' local knowledge of tree provision of ecosystem services to strengthen the emergence of coffee-agroforestry landscapes in southwest China
Source: PLoS One. 2018 Sep 20;13(9):e0204046. doi: 10.1371/journal.pone.0204046 (PMC6147441; doi:10.1371/journal.pone.0204046)
Supplement: S3 Appendix — (DOCX) [file pone.0204046.s003.docx]

# S3 Appendix. Comparison of tree species ranking according to gender and ethnicities


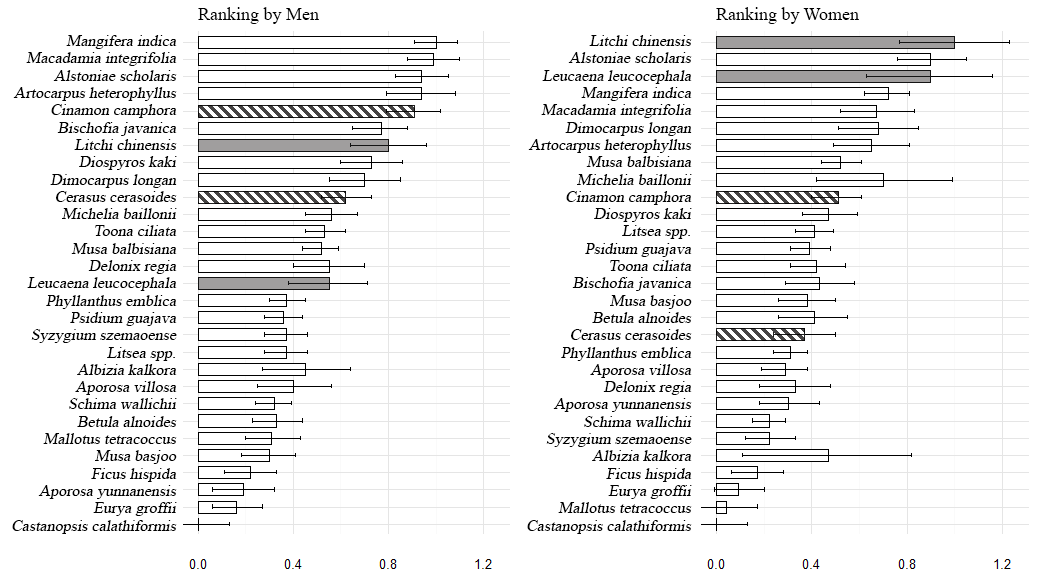


S3 Fig A: Scores and 95% confidence intervals for overall preferences of shade tree species broken down by gender. Grey areas indicate species ranked more highly by female respondents; striped boxes indicate those ranked more highly by male respondents.


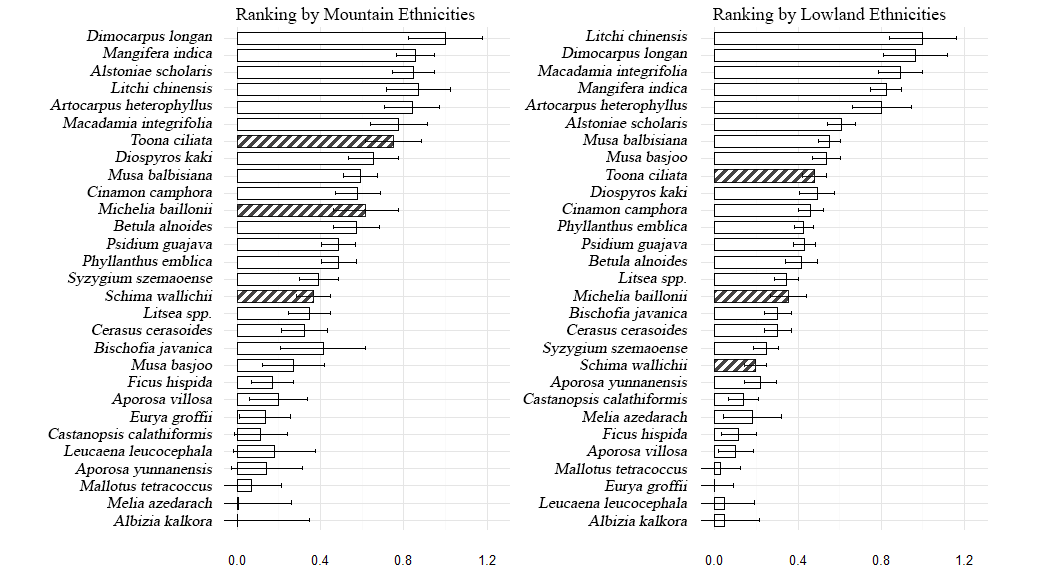


S3 Fig B: Scores and 95% confidence intervals for economic benefits from shade tree species broken down by groups of ethnicities. Grey boxes indicate timber species for which respondents from mountain ethnicities perceived more economic potential than respondents from lowland ethnicities did.
